# BOLD mapping of human epileptic spikes recorded during simultaneous intracranial EEG-fMRI: The impact of automated spike classification

**DOI:** 10.1016/j.neuroimage.2018.09.065

**Published:** 2019-01-01

**Authors:** Niraj K. Sharma, Carlos Pedreira, Umair J. Chaudhary, Maria Centeno, David W. Carmichael, Tinonkorn Yadee, Teresa Murta, Beate Diehl, Louis Lemieux

**Affiliations:** a Department of Clinical and Experimental Epilepsy, UCL Institute of Neurology, Queen Square, London, United Kingdom; b MRI Unit, Epilepsy Society, Chalfont St Peter, Buckinghamshire, United Kingdom; c Department of Experimental Psychology, University of Oxford, Oxford, United Kingdom; d Sensium Healthcare, Milton Park, Abingdon, Oxfordshire, United Kingdom; e Neurology Department, Queen Elizabeth Hospital, University Hospital Birmingham, NHS Foundation Trust, United Kingdom; f National Hospital for Neurology and Neurosurgery, UCLH NHS Foundation Trust, Queen Square, London, United Kingdom; g Epilepsy Unit, Neurology Department, Clinica Universidad de Pamplona, Navarra, Spain; h Developmental Imaging and Biophysics, UCL Institute of Child Health, London, United Kingdom; i Wellcome EPSRC Centre for Medical Engineering, King's College London, St Thomas' Hospital, London, United Kingdom; j Prasat Neurological Institute, Bangkok, Thailand; k National Physical Laboratory, Teddington, Middlesex, United Kingdom

**Keywords:** EEG-fMRI, IED, Intracranial EEG, Automated IED classification, BOLD response, IED, interictal epileptiform discharge, WC, Wave_clus, BOLD, Blood oxygen level dependent, EZ, epileptogenic zone, icEEG, intracranial EEG, CED, continuous epileptiform discharge, SED, single isolated epileptiform discharge

## Abstract

**Objectives:**

Simultaneous intracranial EEG and functional MRI (icEEG-fMRI) can be used to map the haemodynamic (BOLD) changes associated with the generation of IEDs. Unlike scalp EEG-fMRI, in most patients who undergo icEEG-fMRI, IEDs recorded intracranially are numerous and show variability in terms of field amplitude and morphology. Therefore, visual marking can be highly subjective and time consuming. In this study, we applied an automated spike classification algorithm, *Wave_clus* (WC), to IEDs marked visually on icEEG data acquired during simultaneous fMRI acquisition. The motivation of this work is to determine whether using a potentially more consistent and unbiased automated approach can produce more biologically meaningful BOLD patterns compared to the BOLD patterns obtained based on the conventional, visual classification.

**Methods:**

We analysed simultaneous icEEG-fMRI data from eight patients with severe drug resistant epilepsy, and who subsequently underwent resective surgery that resulted in a good outcome: confirmed epileptogenic zone (EZ). For each patient two fMRI analyses were performed: one based on the conventional visual IED classification and the other based on the automated classification. We used the concordance of the IED-related BOLD maps with the confirmed EZ as an indication of their biological meaning, which we compared for the automated and visual classifications for all IED originating in the EZ.

**Results:**

Across the group, the visual and automated classifications resulted in 32 and 24 EZ IED classes respectively, for which 75% vs 83% of the corresponding BOLD maps were concordant. At the single-subject level, the BOLD maps for the automated approach had greater concordance in four patients, and less concordance in one patient, compared to those obtained using the conventional visual classification, and equal concordance for three remaining patients. These differences did not reach statistical significance.

**Conclusion:**

We found automated IED classification on icEEG data recorded during fMRI to be feasible and to result in IED-related BOLD maps that may contain similar or greater biological meaning compared to the conventional approach in the majority of the cases studied. We anticipate that this approach will help to gain significant new insights into the brain networks associated with IEDs and in relation to postsurgical outcome.

## Highlights

• IcEEG-fMRI provides a unique insight into the generators of IEDs.
• Visual IED marking can be highly subjective and time consuming.
• An automated spike classification algorithm, *Wave_clus*, can minimise subjectivity.
• The BOLD maps associated with IEDs classified using *Wave_clus* may commonly have equal or greater biological meaning than those obtained using conventional, visual classification.

## 1 Introduction

Simultaneous EEG-fMRI has been shown to be a useful tool in mapping the regions associated with the generation of interictal epileptiform discharges (IEDs). For example, blood-oxygen level dependent (BOLD) mapping of IEDs detected on scalp EEG can provide added value to the localisation of the EZ (Zijlmans et al., 2007, Moeller et al., 2009; Thornton et al., 2010, 2011, Pittau et al., 2012, An et al., 2013, Coan et al., 2015; Centeno et al., 2017). In one study, patients were reconsidered for surgery after identifying the BOLD correlates of IEDs (Zijlmans et al., 2007) and others have suggested its potential use in predicting postsurgical outcome (Thornton et al., 2010, An et al., 2013, Coan et al., 2015; Centeno et al., 2017). In these studies, IED-related BOLD changes have been found to be located in proximity, but also often remote from the EZ suggesting that their generators can involve a widespread network. This may partly reflect the area of synchronous neural activity required for their detection on scalp EEG (Tao et al., 2005). However, the sensitivity of detecting IEDs during an fMRI scan can be a limitation for scalp EEG-fMRI studies (Aghakhani et al., 2015; Salek-Haddadi et al., 2006). In contrast, the sensitivity of intracranial EEG (icEEG) to IEDs is much greater, due to the lack of attenuation and spatial integration from the scalp and the skull (Carreno and Luders, 2001), which has led to the implementation of simultaneous icEEG and fMRI acquisitions (icEEG-fMRI) in an effort to better understand the haemodynamic changes associated with epileptic activity (Carmichael et al., 2012; Boucousis et al., 2012).

To date, there have only been three studies investigating the whole-brain haemodynamic correlates of IEDs detected on icEEG (Vulliemoz et al., 2011; Cunningham et al., 2012; Aghakhani et al., 2015). In these studies, the IEDs were detected and classified by an EEG reviewer in the usual visual manner and a general linear model (GLM) was used to map the BOLD correlates of each IED class. From a theoretical viewpoint, in order to create the optimal model of the IED-related BOLD changes, it is important that once the IEDs have been detected, that they be classified so that each regressor represents the activity of a specific neuronal population to the exclusion of other generators (Liston et al., 2006). In the conventional approach the classification of IEDs generally requires the reviewer to distinguish different IEDs based on their field distribution: essentially the EEG channels in which they occur (Gotman, 1999; James et al., 1999). However as noted above, the high sensitivity of icEEG can result in much more abundant IEDs than in scalp recordings, and often more varied in their morphology and distribution than on scalp EEG (Spencer et al., 2007). Indeed, the detection and classification of IEDs on icEEG by clinical neurophysiologists can be time consuming and is unreliable (Dümpelmann and Elger, 1999; Barkmeier et al., 2012; Gaspard et al., 2014; Sharma et al., 2017). Furthermore, the incorrect and inconsistent markings of IEDs have been shown to result in an excess of false positive and false negative BOLD clusters (Flanagan et al., 2009).

Automated algorithms for the analysis of IEDs on icEEG have been designed with the principal aim of reducing the subjectivity of EEG marking (Dümpelmann and Elger, 1999; Bourien et al., 2005; Valenti et al., 2006; Brown et al., 2007; Barkmeier et al., 2012; Gaspard et al., 2014). Such methods are able to detect IEDs but do not exploit the relationship between the activity across channels. Given that IED field distribution is an important consideration in the way IEDs are typically classified visually, we favour automated schemes that incorporate the waveforms across several channels (Hufnagel et al., 2000; Bourien et al., 2005; Pedreira et al., 2014; Sharma et al., 2017). To our knowledge, the only such classification scheme evaluated by comparison with multiple observers was Sharma et al. (2017). This study validated an automated neuronal spike classification algorithm, *Wave_clus* (WC) (Quiroga et al., 2004; Pedreira et al., 2014) for the automated classification of icEEG IEDs (Sharma et al., 2017), by formally demonstrating that the WC results fall within the observed inter-rater variability for three expert EEG reviewers.

In the present work, our aim was to use this automated IED classification algorithm (WC) (Sharma et al., 2017) to determine whether the models of the IED-related BOLD changes obtained based on the resulting IED classification are comparable to those obtained using the traditional, visual approach. This follows our previous work on the evaluation of the value of the automated classification of IED on scalp EEG-fMRI based on their BOLD correlates (Pedreira et al., 2014); In essence we use the fMRI data as an additional, independent means of assessing the IED classifications’ biological value. To investigate this, we analysed icEEG-fMRI data collected from patients who subsequently underwent resective surgery that resulted in a good postsurgical outcome, thereby providing some confirmation of the epileptogenic zone (EZ). We compared the BOLD maps obtained using two general linear models: one derived from the IEDs classified automatically and the other derived from the IEDs classified using the conventional visual method; this comparison was made in terms of localisation of the IED-related BOLD changes in relation to the confirmed EZ. We hypothesised that an automated approach to IED classification can result in more or equally concordant, and therefore biologically meaningful, BOLD maps.

## 2 Methods

### 2.1 Patients

Eight patients (6 males; age range: 32–42 years) who underwent simultaneous intracranial EEG-fMRI at the National Hospital for Neurology and Neurosurgery (UCLH NHS Foundation Trust, Queen Square, London, UK) were included in this study (Table 1). These patients were part of a group of 19 who underwent icEEG-fMRI; we retrospectively selected all those who subsequently underwent resective surgery with a good postsurgical outcome (ILAE 1; Wieser et al., 2001) (no seizure for at least 3 years post-resection). The patients gave informed written consent for participation in this study, which was approved by the joint research ethics committee of the National Hospital for Neurology and Neurosurgery and UCL Institute of Neurology, Queen Square, London, UK.

**Table 1:** Summary of non-invasive electro-clinical information.

Patient	Age/Sex	Age at seizure onset	Scalp EEG	MRI findings	Other non-invasive investigations
1	36/M	12	Spikes: L fronto-central Seizure: Regional central	FCD L posterior SFG + MFG	PET: normal Ictal SPECT: L frontal lobe
2	37/M	9	Spikes: Regional L temporal-frontal Seizure: Regional L fronto-central	FCD L posterior MFG	PET: normal
3	41/F	7	Spikes: Regional L inferior frontal/orbital frontal Seizure: Regional L frontal	FCD L IFG	PET: L frontal hypometabolism
4	34/M	7	Spikes: Regional L and R temporal regional Seizure: Regional L temporal	L HS	None
5	37/M	16	Spikes: Regional R frontal, bi frontal and L fronto-temporal Seizure: Regional frontocentral	Non-lesional	PET: R frontal hypometabolism
6	44/M	8	Spikes: Regional R centro-parietal Seizure: Central fast activity	L HS^a^	PET: R parietal and posterior frontal hypometabolism Ictal SPECT: bi frontocentral and R insula hyperperfusion MEG: R temporo-occipital and frontocentral spikes
7	32/F	3	Spikes: None Seizure: Regional L frontocentral	FCD L superior frontal sulci	PET: L SFG hypometabolism Ictal SPECT: L frontal and insular hyperperfusion MEG: No spikes recorded
8	33/M	7	Spikes: Regional right anterior parietal Seizure: Regional R postcentral	FCD R frontoparietal	None

Abbreviations M: Male; F: Female; FLE: Frontal lobe Epilepsy; PLE: Parietal lobe epilepsy; R: Right; L: Left; FCD: Focal cortical dysplasia; HS: Hippocampal sclerosis; SFG: Superior frontal gyrus; IFG: Inferior frontal gyrus; MFG: Middle frontal gyrus.

a Incidental finding.

At the time of participation in this study all patients were undergoing invasive icEEG recordings for clinical purposes to assess their suitability for curative surgery. The need for invasive EEG recordings had been established in a multidisciplinary meeting to delineate the ictal onset zone and/or to perform direct electro-cortical stimulation for functional mapping. Prior to the invasive EEG recordings, the patients had undergone standard pre-surgical evaluation including long-term video-scalp EEG monitoring, structural MRI and other investigations such as positron emission tomography (PET), magnetoencephalography (MEG) or ictal single photon emission computed tomography (ictal SPECT) when available.

### 2.2 Simultaneous icEEG-fMRI acquisition

Between 31 and 91 electrode contacts were implanted in each patient with grid, strip and depth electrodes or a combination thereof (all Ad-Tech subdural epilepsy or Spencer Probe depth electrodes; Ad-Tech Medical Instrument Corporation, Racine, WI, USA); see Table 2. The electrodes were connected to an MR-compatible EEG amplifier system (BrainAmp MR+; Brain Products, Gilching, Germany). In accordance with our icEEG-fMRI protocol (Carmichael et al., 2012), echo planar images (EPI) were acquired using a 1.5 T Siemens Avanto scanner (Erlangen, Germany) with a standard birdcage transmit/receive head-coil. Depending upon patient comfort inside the scanner and time constraints either one (for patients 1, 2, 3, 4) or two (patients 5, 6, 7, 8) useable 10-min resting-state EPI time series was obtained; a T1-weighted structural scan was also acquired. The icEEG signals were recorded at a sampling rate of 5 kHz. In the case of patient 3 the scanning-related artefacts on icEEG during the first EPI sequence could not be corrected satisfactorily due to a technical problem at acquisition; therefore, only the second EPI sequence was included in this study. Patient 4 had a subclinical seizure during one of two EPI series (see Chaudhary et al., 2016 for a report on the analysis of this data). Therefore, 2/12 sessions were not included in this study.

**Table 2:** **Details of icEEG implantation, invasive and surgical clinical findings, and icEEG during fMRI data.**The anatomical descriptions and electrode labels were taken from the clinical reports.

Patient	icEEG implantation		Clinical findings		icEEG-fMRI		
	Localisation	Electrodes (*labels*)^a^	EZ localisation	Surgical outcome (# months post-surgery)	Number of 10-min fMRI sessions	Number ofIZ1 IEDs on icEEG during fMRI identified by H1	Channels of Interest (COI) for WC classification
**1**	• L superior, middle and inferior frontal gyri. • L precentral gyrus • L central sulcus and part of postcentral sulcus. • L superior frontal sulcus • L postcentral regions	• One 8x8-contacts grid (*G*) • Two 4-contacts depths (*DA* and *DP*) • One 2x8-contacts grid (*GA*)	L Posterior SFG, MFG, SMA	ILAE 1 (71)	1	590	G4 G5 G13 G20 G21 G22 G23 G29 DP2 DP3
**2**	• L frontal lobe (laterally and inferiorly) • L middle and inferior frontal gyrus • L frontal pole	• One 8x8-contacts grid (*GA*) • One 2x8-contacts grid (*GD*) • Two 6-contacts depths (*DA* and *DP*) • Two 6-contacts strips (*GC* and *GB*)	L IFG, MFG and lateral orbitofrontal	ILAE 1 (57)	1	892	GA50 GA51 GA52 GA53 DA4 DA5
**3**	• L inferior and middle frontal gyrus • L frontal pole	• One 8x8-contacts grid (*G1*) • One 2x8-contacts grid (*G2*) • Two 6-contacts depths (*DA*and *DP*) • One 6-contacts strip (*S1*)	L anterior IFG and MFG	ILAE 1 (68)	1	1035	DA3 DA4 G1_26 G1_34 G1_35 G1_36 G1_42 G1_43 G1_44 G1_50 G2_5 G2_6 G2_13 G2_14
**4**	• R and L amygdalae • R and L hippocampi	• Five 6-contacts depths (*LA*, *LAH*, *LPH*, *RA* and *RH*)	L anterior temporal lobe	ILAE 1 (87)	1	572	LA2 LA3 LH1 LH2 LP1 LP2 RA1 RA2 RA3 RH1
**5**	• R anterior and posterior mesial frontal lobe • R orbitofrontal lobe • R anterior and posterior supplementary sensorimotor areas • R frontal pole.	• Three 16-contacts depths (5 mm spacing) (*AM*, *PM* and *PMFG*) • Four 12-contacts depths (5 mm spacing) (*FP*, *ASMA*, *PSMA* and*IFG*) • One 10-contacts depth (*FOF*)	R anterior orbitofrontal	ILAE 1 (56)	2	223	AM2 AM3 AM4 Fp1 Fp2 Fp3 Fp4 PMFG3 PMFG4 IFG9
**6**	• R anterior and posterior insula • R anterior and posterior supplementary motor areas • R anterior, middle and posterior cingulum	• Two 6-contacts depths (*ASMA* and *PSMA*) • Three 8-contacts depths (*AC*, *MC* and *PC*).	R SMA and SFG	ILAE 1 (72)	2	733	ASMA1 ASMA2 ASMA3 PSMA1 PSMA2 PSMA3 PC4 PC5 AI5 AI6
**7**	• L superior and middle frontal gyrus • L frontal pole	• One 8x8-contacts grid (*GA*) • One 2x8-contacts grid (*GB*) • One 4x8-contacts grid (5 mm spacing) (*GC*) • One 8-contacts strip (*SF*).	L posterior SFG (lateral and medial)	ILAE 1 (56)	2	755	SF5 SF6 SF7 SF8 GB4 GB5 GB6 GB7
**8**	• R fontoparietallobe (laterally and inferiorly)	• One 8x8-contacts grid (*G*) • Two 6-contacts depths (*D1* and *D2*)	R frontoparietal	ILAE 1 (48)	2	3505	D1_3 D1_4 G22 G23 G29 G30 G31 G37 G38 G39 G40 G45 G46 G47

Abbreviations R: right; L: left; A: anterior; P: posterior; SMA: supplementary (sensory)motor area; C: cingulum; IFG: inferior frontal gyrus; MFG: middle frontal gyrus; SFG: superior frontal gyrus.

a All with 10-mm spacing unless indicated.

### 2.3 icEEG pre-processing and analysis

Two IED classification strategies were employed for subsequent BOLD mapping using general linear modelling (GLM): firstly, conventional visual IED classification (Vulliemoz et al., 2011); and secondly, automated IED classification using a version of *WC* (Quiroga et al., 2004) adapted specifically for epileptic discharges on icEEG (Sharma et al., 2017).

Pathological EEG patterns commonly found in invasive recordings include individual IEDs (spikes and sharp waves), repetitive IEDs (polyspikes) and paroxysmal fast activity (PFA) (Widdess-Walsh et al., 2007). Individual and repetitive IEDs can occur as single isolated epileptiform discharges (SED) or continuous epileptiform discharges (CED) (Palmini et al., 1995; Turkdogan et al., 2005). In two patients (# 3 and 8), there were CED with more than 1 IED/sec, which precluded the use of WC for their classification, due to the close proximity in time of the discharges, which causes difficulties in isolating them within the time window used in the *WC* process (see Sharma et al., 2017). In another patient (#4), polyspikes were observed that had the same field as individual IED; these were not classified using WC.

Prior to IED detection and classification, offline, average template subtraction-based, correction for MR scanning artefacts was applied to the EEG (Allen et al., 2000) and the resulting signals were down sampled to 250 Hz, and band-pass filtered (2–70 Hz).

#### 2.3.1 Visual IED marking and classification

EEG reviewer ‘H1’ (UJC) marked all clear or suspected individual SED and CED using the *BrainVision Analyser* software (Brain Products, Germany), based on their morphology and field using two types of event markers: point markers for individual IEDs, and onset and offset markers for repetitive IEDs (CED) and PFA.

#### 2.3.2 Automated (*Wave_clus*) IED classification

Following our previous work, *WC* (Pedreira et al., 2014; Sharma et al., 2017) was used to automatically classify the IEDs identified by H1 (see above): SEDs and CEDs (<1 IED/sec). In summary, *Wave_clus* is an automated neuronal spike classification algorithm that identifies and exploits small but consistent differences across multiple waveforms using wavelet decomposition and a superparamagnetic clustering algorithm (Quiroga et al., 2004). For the purpose of automatically classifying icEEG IEDs, this process was carried out over the event waveforms captured on between 8 and 14 icEEG channels (the channels of interest) in which the IEDs were noted in the clinical EEG report as being most frequent and prominent (see Sharma et al., 2017 for a more detailed description). The result of this process was a set of IED classes (and corresponding labels) with every IED assigned to one of the classes. Polyspikes with the same field as a given *Wave_clus* class were subsequently added to this class.

#### 2.3.3 Electro-clinical labelling of IED classes

In order to facilitate the interpretation and presentation of the findings, the IED classes were also labelled based on two criteria: 1) extent of their field distribution and 2) spatial relationship to the EZ.

##### 2.3.3.1 Extent of field distribution

For field distribution, IEDs were labelled according to their spatiotemporal localisation and distribution across the implanted electrodes (Lüders et al., 2006) as either:

• Focal: if they involved up to 4 contiguous electrode contacts,
• Regional: if they involved 5–10 contiguous electrode contacts,
• Widespread: if they involved more than 10 contiguous electrode contacts, or:
• Non-contiguous: if they had a focal or regional field but also extended to non-contiguous electrode contacts

##### 2.3.3.2 Relationship to the EZ

IED classes were also labelled according to their spatial relationship to the EZ. All IED classes recorded in, and limited to, the brain area overlapping the EZ were labelled as IZ1 (‘primary irritative zone’). All IED classes extending to brain areas outside the EZ were labelled as IZ2 (‘secondary irritative zone’) (Bettus et al., 2011). In this study we focused on the IZ1 IED, as they are the most clinically relevant (Cunningham et al., 2012; Vulliemoz et al., 2011, An et al., 2013).

### 2.4 fMRI data analysis

As mentioned previously, we created two GLMs to map the IED-related BOLD changes using the Statistical Parametric Mapping software (SPM8; https://www.fil.ion.ucl.ac.uk/spm/software/spm8/) for each patient: one based on the visually classified IEDs (GLM1) and the other based on the automated IED classification (GLM2). This is in line with our previous study on the application of *Wave_clus* to scalp EEG-fMRI (Pedreira et al., 2014).

Each IED class was modelled as a separate effect of interest. For the CED with more than 1 IED/sec which were not classified using WC (one class for patients 3 and 8, as described above) the visual classification was used as an effect of interest in GLM1 and GLM2; furthermore, in patient 4 visually identified polyspikes (which could not be classified automatically) were added to a WC class that contained individual IED with the same field distribution, for inclusion in GLM2.

Individual IED markers were represented as a zero-duration stick function and polyspikes and paroxysmal fast activity were represented as variable duration blocks and convolved with the canonical hemodynamic response function, and its temporal and dispersion derivatives (canonical HRF + TD + DD).

The first two volumes of the fMRI time series data were discarded to account for the T1-saturation effect; slice timing correction, scan realignment to the mean and spatially smoothed using an isotropic Gaussian kernel of 8 mm Full Width Half Maximum (FWHM) were employed (Friston et al., 1994). For patients who underwent two EPI series (patients 5, 6, 7, 8), these were included in a single GLM as separate sessions.

Twenty-four inter-scan realignment parameters - 6 realignment parameters from image pre-processing and a Volterra expansion of these (Friston et al., 1996) were included in the GLM as confounds to account for motion-related effects. We then applied a robust weighted least squares toolbox (Diedrichsen and Shadmehr, 2005) to reduce the influence of potential physiological and other sources of noise and artefacts.

For each IZ1 IED class, the presence of significant BOLD changes was assessed over the whole brain using a F contrast across the canonical HRF + TD + DD regressors, at a statistical threshold of *p* < 0.001 (uncorrected for multiple comparisons) and a cluster size threshold of 5 contiguous voxels (Grouiller et al., 2011; Centeno et al., 2017). The resulting SPMs were co-registered with pre- and post-surgical T1-weighted MRI scans using the rigid-body registration tool in SPM.

#### 2.4.1 Concordance: localisation of the IED-related BOLD maps in relation to EZ

For each IZ1 IED class, we evaluated the spatial concordance with the EZ, irrespective of the sign of BOLD change considering that both increases and decreases have been found in the epileptogenic zone (Thornton et al., 2011; Chaudhary et al., 2012; Pittau et al., 2013). Similarly to our previous work (Coan et al., 2015) the concordance of each IED-related BOLD map with the EZ was assessed as either:

• *Concordant (C):* when one or more BOLD clusters overlapped with the area of surgical resection or were within up to 2 cm (Cartesian distance, within the same lobe) of the resection margin.
• *Discordant* (*D*): all clusters were remote (different lobe or opposite hemisphere) from the EZ.

Although the above assessment does not consider the location of the BOLD cluster containing the global (statistical) maximum (GM; as automatically calculated by SPM8), we report it as it is of some interest in view of its previous use in some studies of fMRI mapping of IED recorded on scalp EEG (Moeller et al., 2009; Thornton et al., 2010; Pittau et al., 2012; An et al., 2013).

For each patient we calculated the proportion of *C* maps for GLM1 and GLM2: (number of *C* maps)/(number of maps); the Wilcoxon signed-rank test was used at the group level.

## 3 Results

Six of the eight patients were diagnosed with frontal lobe epilepsy, one had temporal lobe epilepsy and one had parietal lobe epilepsy (see Table 1). The mean number of months they were seizure free post resective surgery was 64 months (*SD*: 12mo; range: 48–87mo) (see Table 2). Across all subjects, the mean number of IED events detected by H1 was 1206 (*SD*: 989; range: 460–3567; Table 2) and the mean number of events classified by WC was 1105 (*SD*: 915; range: 277–3246; Table 3). In the following we summarise the IED classes obtained by the EEG reviewer and the automated algorithm.

**Table 3:** Visual and *Wave_clus* IZ1 IED classes and BOLD map concordance.

Patient	GLM1 (Visual)				GLM2 (Automated)				
	IED class (number)	Field distribution	Concordance	% Concordance	Spike class (number)	Field distribution		Concordance	% Concordance
1	G4,5 (70)	Focal	Y	100	G23 (127)	Focal		Y	100
	G12-15 (30)	Focal	Y		G4_5_13_21_29_DP (131)	Regional		Y	
	G4-6 + G12,13 + G22-24 + G28-30 (60)	Regional	Y		G4_5_29 (78)	NC		Y*	
	G12-15 + G21-24 + DP2-4 (218)	Regional	Y*		G13_20_21_DP (152)	Focal		Y	
	G4-8 +G12-15 + G20-24 + G28-30 + DP2-4 (212)	Widespread	Y*						
2	DA3-6 (423)	Focal	Y	66	D4_5 (498)		Focal	Y	66
	DA4,5 + GA51 (261)	Focal	N		D4_5_GA50_51_52 (106)		Regional	N	
	DA2-6 + GA49-54 (208)	Regional	Y		D4_5_GA51 (156)		Focal	Y	
3	DA3,4 (770)	Focal	N	50	DA3_4 (770)		Focal	N	0
	DA3,4 + G1 18,27,35,43 (265)	Regional	Y*		DA3_4_G1_26 G1_34 G1_35 G1_36 G1_42 (75)		Regional	N	
4	LAH1,2 + LPH1,2 + LA3,4 (60)	Regional	Y	50	LA2_LAH1_2 (360)		Focal	Y	100
	LAH1-2 (359)	Focal	Y		LAH1_2_LPH1(112)		Focal	Y*	
	LA3-4 (57)	Focal	N						
	LPH1-2 (359)	Focal	N						
5	FP2-4 (142)	Focal	Y	50	FP4 (333)		Focal	Y	100
	FP2-4 + AM2-4 (81)	Regional	N		AM2-4 + FP1-4 (591)		Regional	Y	
6	PSMA1-3 (211)	Focal	Y	100	ASMA1-2 + PSMA2-3 (364)		Focal	Y	100
	ASMA1-3 (46)	Focal	Y		PSMA2-3 (250)		Focal	Y	
	ASMA1-3 + PSMA1-3 (476)	Regional	Y						
7	SF5-7 (168)	Focal	N	75	SF_GB (68)	NC		Y	100
	GB4-6 + 14–16 (90)	Regional	Y		SF6_7 (177)	Focal		Y	
	GC5-16 (474)	Regional	Y		SF8 (32)	Focal		Y	
	SF5-7 + GB5-8 + GC5,10,11,12,15,16 (23)	NC	Y						
8	D1 (43)	Focal	Y	78	D2_3_4 (2481)	Focal		Y*	83
	D2 (2481)	Focal	Y*		G38-39-40-46-47 (256)	Regional		Y*	
	G23 (83)	Focal	Y		D1_3_4 G37-38-39 (184)	Regional		Y	
	G31 (72)	Focal	Y		D1_3_4 (75)	Focal		Y*	
	G36 (209)	Focal	Y		G45-46 (128)	Focal		N	
	G38 (226)	Focal	Y*		D1_3_4 G22_23_29_30_31_37_38 (66)	Widespread		Y	
	G44 (140)	Focal	N						
	G45 (127)	Focal	Y						
	G47 (124)	Focal	N						

Note: * = BOLD cluster in area of resection is the global maximum (GM).

### 3.1 IED detection and visual and automated classification

#### 3.1.1 Visual classification

Thirty-three classes were IZ1 IEDs, with at least two in every patient: patient 1, *N* = 5 (Focal: 2, Regional: 2, Widespread: 1); patient 2, *N* = 3 (Focal: 2, Regional: 1); patient 3, *N* = 2 (Focal and regional); patient 4, *N* = 4 (Focal: 3, Regional: 1), patient 5, *N* = 2 (Focal: 1, Regional: 1), patient 6, *N* = 3 (Focal: 2, Regional: 1); patient 7, *N* = 4 (Focal: 1, Regional: 2, Non-contiguous: 1), patient 8, *N* = 9 (Focal: 9) (see Table 3). The mean number of IZ1 IEDs was 1039 (*SD*: 1024; range: 223–3505). See Table 2 (Supplementary Table 1 for the IZ2 classes).

#### 3.1.2 *Wave_clus* classification

Twenty-three classes were labelled as IZ1 IEDs with at least two in every patient: patient 1, *N* = 4 (Focal: 2, Regional: 1, Non-contiguous: 1); patient 2, *N* = 3 (Focal: 2, Regional = 1); patient 3, *N* = 1 (Regional); patient 4, *N* = 2 (Focal), patient 5, *N* = 2 (Focal and Regional); patient 6, *N* = 2 (Focal); patient 7, *N* = 3 (Focal: 2, Non-contiguous: 1); patient 8, *N* = 6 (Focal: 2, Regional: 3, Widespread: 1). The mean number of IZ1 IED was 946 (*SD*: 931; range: 277–3190). See Table 3 (Supplementary Table 1 for the IZ2 classes.)

Two out of three CED IZ1 IED classes (patient 3: 1 Focal IED class; *N* = 770 and patient 8: 1 Focal IED class; *N* = 2481) were not classified using *Wave_clus* as they occurred >1 per sec.

### 3.2 Concordance of IZ1 IED-related BOLD changes: visual (GLM1) vs *Wave_clus* (GLM2) classifications

There was no significant difference in the proportion of *C* maps (Wilcoxon signed rank test: *Z* = −0.96, *p* = 0.3) between GLM1 and GLM2 across the group; see Table 3. For GLM1, 75% (24/32) of the BOLD maps were *C*; 83% (20/24) of GLM2 BOLD maps were *C*. Four patients showed greater concordance for GLM2 compared to GLM1 (patient 4, 5, 7 and 8), three patients had equal concordance for GLM1 and GLM2 (patient 1, 2 and 6), and one patient showed lower concordance (patient 3).

In relation to IED field, for GLM1 there was a degree of concordance in 14/18 of Focal, 8/8 of Regional, 1/1 of Widespread, and 1/1 of Non-contiguous IED classes. For GLM2, there was a degree of concordance in 14/16 of Focal, 4/6 of Regional and 2/2 of Non-contiguous classes. See Supplementary Tables 2 and 3 for more complete descriptions of the GLM1 and GLM2 BOLD maps respectively.

#### 3.2.1 Case reports

To better illustrate the range of results obtained we present four case reports: two in which GLM2 showed greater BOLD concordance (patients 4 and 5), one in which concordance was the same for GLM1 and GLM2 (patient 6) and one in which the maps obtained from the automated classification were less concordant (patient 3). We report only on the results for the IZ1 IED.

##### 3.2.1.1 Patient 4: improved concordance

This patient was diagnosed with temporal lobe epilepsy and the EZ was located in the left anterior temporal lobe.

###### 3.2.1.1.1 IED classification

Reviewer H1 detected 1216 IEDs.

**Fig. 1:**
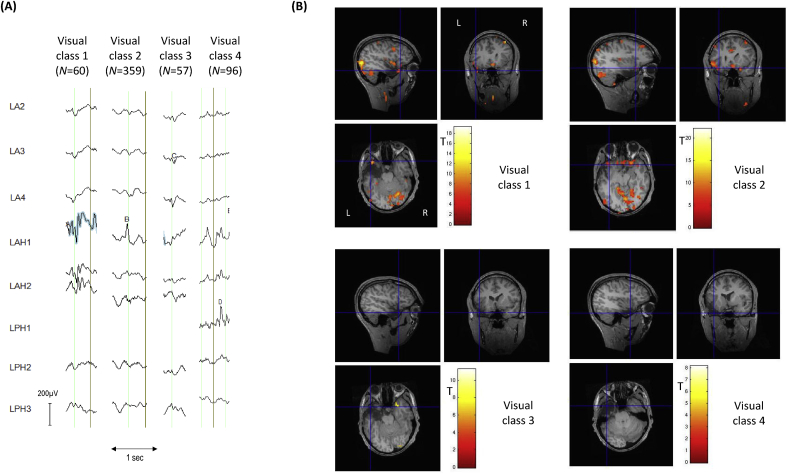
**Patient 4: Visual IZ1 IED classes and BOLD maps (GLM1)** (A) Samples for each of the four IED classes identified by EEG reviewer H1; the blue highlight is automatically generated by the *Brain Vision Analyzer* EEG display software to show event marking; (B) IED-related BOLD maps for each of the visual IED classes, superimposed on the patient's co-registered postoperative structural MRI. The maps' concordances were: Visual class 1: concordant; Visual class 2: concordant; Visual class 3: discordant; Visual class 4: discordant. Cross-hair placement: for the concordant maps, within one of the BOLD clusters within, or overlapping with, the EZ, for discordant maps: within the EZ.

*Visual classification*: Reviewer H1 classified the IEDs into six classes, four of which were IZ1 (classes 1–4) comprising a total of 572 IEDs; class 1 consisted of regional spikes (*N* = 60); classes 2, 3 and 4 consisted of focal spikes (*N* = 359, 57 and 96 respectively; see Fig. 1). Class 2 included polyspikes.

**Fig. 2:**
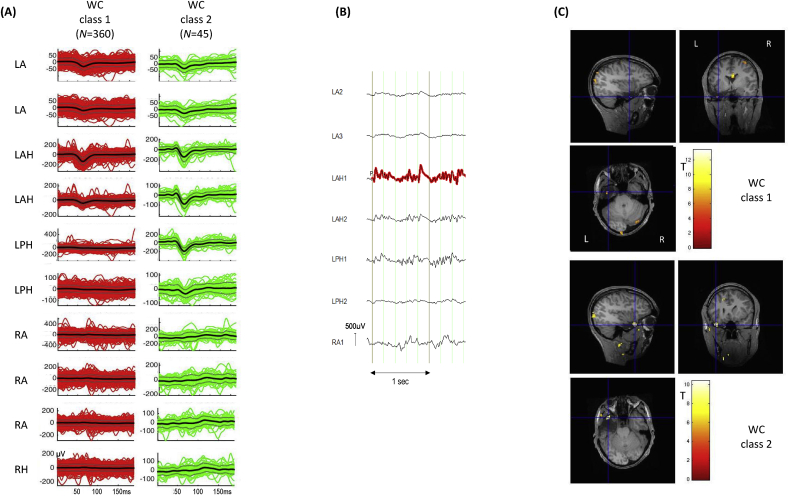
**Patient 4: *Wave_clus* IZ1 IED classes and BOLD maps (GLM2)** (A) Butterfly plots for each of the two IED classes obtained using *WC*. The colored traces (red for WC class 1; green for WC class 2) represent the individual events, the thick black lines the mean traces and grey lines, the standard error. (B) Example of visually classified polyspikes, 67 of which were added to *Wave_clus* class 2; the red highlight is automatically generated by the *Brain Vision Analyzer* EEG display software to show event marking. (C) IED-related BOLD maps for the IZ1 WC classes, superimposed on the patient's co-registered postoperative structural MRI. The maps' concordances were: WC class 1: concordant; WC class 2: concordant. Cross-hair placement: for the concordant maps, within one of the BOLD clusters within, or overlapping with, the EZ, for discordant maps: within the EZ.

*Automated classification*: The automated algorithm classified the IEDs into four classes, two of which were in IZ1 (classes 1 and 2) totalling 405 focal spikes (*N* = 360 and 45, respectively; see Fig. 2).

###### 3.2.1.1.2 BOLD results

GLM1: the concordance for the three IZ1 classes was: *C* for classes 1 and 2, and *D* for classes 3 and 4. For classes 1 and 2 the global maximum was located in the left lateral occipital lobe; the GM was in the right superior frontal gyrus for class 3 and in the left orbital frontal cortex for class 4.

GLM2: 67 visually classified polyspikes were found to match the field distribution and morphology of WC class 2 and therefore included in GLM2 in the same effect as WC class 2 (for a total of 112 IEDs). The concordance for the two IZ1 classes was: *C*. For class 1 the global maximum was located in the anterior cingulate cortex and for class 2 the global maximum was located within the EZ.

##### 3.2.1.2 Patient 5: improved concordance

This patient was diagnosed with frontal lobe epilepsy and the EZ was located in the right anterior orbitofrontal region.

###### 3.2.1.2.1 IED classification

Reviewer H1 detected 1140 IEDs.

**Fig. 3:**
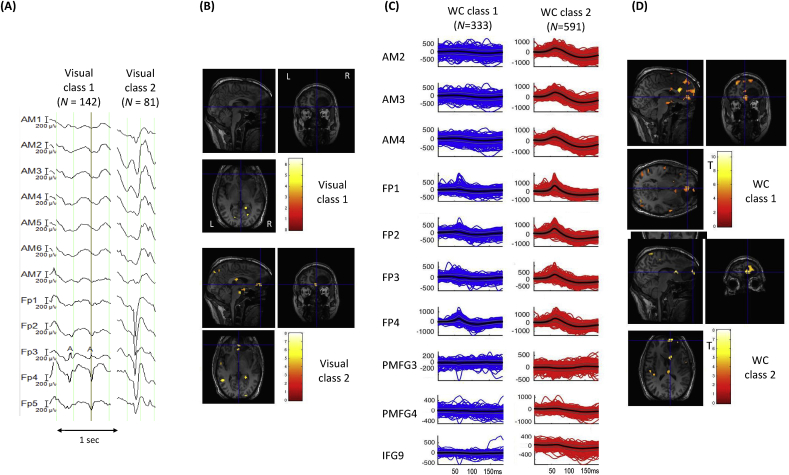
**Patient 5: Visual and *Wave_clus* IZ1 IED classes and BOLD maps (GLM1 and GLM2)** (A) Samples for each of the two IED classes identified by EEG reviewer H1. (B) IED-related BOLD maps for the two IZ1 visual classes, superimposed on the patient's co-registered postoperative structural MRI. The maps' concordances were: Visual class 1: Concordant; Visual class 2: Discordant. (C) Butterfly plots for each of the two IED classes obtained using WC. The colored traces (blue for WC class 1, red for WC class 2) represent the individual events, the thick black lines the mean traces and grey lines, the standard error. (D) IED-related BOLD maps for the two IZ1 WC classes, superimposed on the patient's co-registered postoperative structural MRI. The maps' concordances were: WC class 1: concordant; WC class 2: concordant. Cross-hair placement: for the concordant maps, within one of the BOLD clusters within, or overlapping with, the EZ, for discordant maps: within the EZ.

*Visual classification*: Reviewer H1 classified the IEDs into five classes, two of which were labelled IZ1 (classes 1 and 2) comprising a total of 223 IEDs; class 1 consisted of focal spikes *N* = 142), while class 2 consisted of regional spikes; see Fig. 3.

*Automated classification*: The automated algorithm classified the IEDs into three classes, two of which were labelled IZ1 (classes 1 and 2) comprising a total of 924 IEDs; class 1 consisted of focal spikes (*N* = 333) and class 2 consisted of regional spikes (*N* = 591).

###### 3.2.1.2.2 BOLD results

*GLM1*: the degree of concordance for the two IZ1 classes was: *C* for class 1 and *D* for class 2, and the global maximum was located in the right mesial occipital lobe for both.

*GLM2*: the degree of concordance for the two IZ1 classes was: *C* for classes 1 and 2. For IED class 1, the global maximum was located in the right occipital lobe, and for IED class 2 it was located in the left superior parietal lobe.

##### 3.2.1.3 Patient 6: no change in concordance

This patient was diagnosed with frontal lobe epilepsy and the EZ was located in the right supplementary motor area and right superior frontal gyrus.

###### 3.2.1.3.1 IED classification

Reviewer H1 detected 1033 IEDs.

**Fig. 4:**
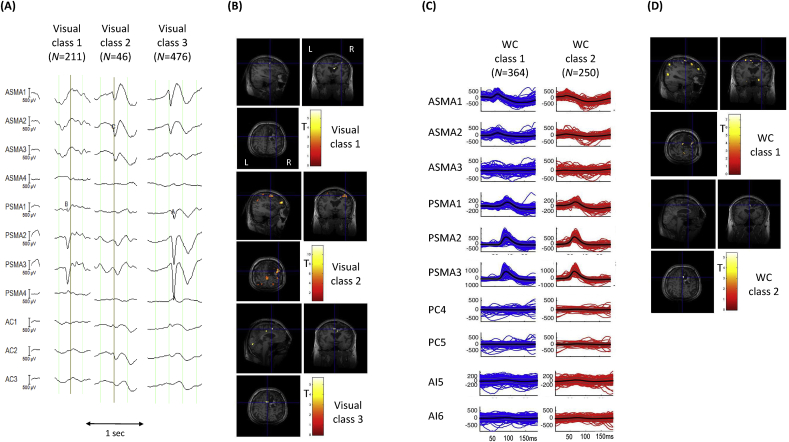
**Patient 6: Visual and *Wave_clus* IZ1 IED classes and BOLD maps (GLM1 and GLM2)** (A) Samples for each of the three IED classes identified by EEG reviewer H1. (B) IED-related BOLD maps for the three IZ1 visual classes, superimposed on the patient's co-registered postoperative structural MRI. The maps' concordances were: Visual class 1: Concordant; Visual class 2: Concordant; Visual class 3: Concordant. (C) Butterfly plots for each of the two IED classes obtained using WC. The colored traces (blue for WC class 1, red for WC class 2) represent the individual events, the thick black lines the mean traces and grey lines, the standard error. (D) IED-related BOLD maps for the two IZ1 WC classes, superimposed on the patient's co-registered postoperative structural MRI. The maps' concordances were: WC class 1: concordant; WC class 2: concordant. Cross-hair placement: for the concordant maps, within one of the BOLD clusters within, or overlapping with, the EZ, for discordant maps: within the EZ.

*Visual classification*: Reviewer H1 classified the IEDs into five classes, three of which were labelled IZ1 comprising a total of 733 IEDs (classes 1–3); classes 1 and 2 consisted of focal spikes (*N* = 211 and 46, respectively), while class 3 consisted of regional spikes (*N* = 476); see Fig. 4.

*Automated classification*: The automated algorithm classified the IEDs into three classes, two of which were labelled IZ1 (classes 1 and 2) and consisted of focal spikes (*N* = 364 and 250, respectively for a total of 614).

###### 3.2.1.3.2 BOLD results

*GLM1*: the concordance for the three IZ1 classes was *C*. For class 1 the global maximum was located in the left orbital frontal cortex, for class 2 the global maximum was located in the right inferior frontal gyrus and for class 3 the global maximum was located in the right fronto-temporal lobe.

*GLM2*: the degree of concordance for the two IZ1 IED classes was *C*. For class #1 the global maximum was located in the left frontal pole, and in the right posterior temporal lobe for class 2.

##### 3.2.1.4 Patient 3: decreased concordance

This patient was diagnosed with frontal lobe epilepsy and the EZ was in the left anterior inferior and middle frontal gyri.

###### 3.2.1.4.1 IED classification

Reviewer H1 detected 1230 IEDs.

**Fig. 5:**
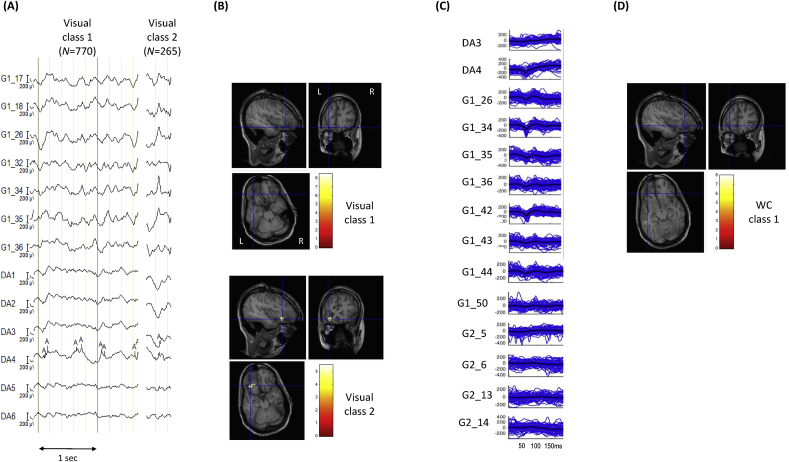
**Patient 6: Visual and *Wave_clus* IZ1 IED classes and BOLD maps (GLM1 and GLM2)** (A) Samples for each of the two IED classes identified by EEG reviewer H1. Visual class 1 consists of CED. (B) IED-related BOLD maps for the two IZ1 visual classes, superimposed on the patient's co-registered postoperative structural MRI. The maps' concordances were: Visual class 1: Discordant; Visual class 2: Concordant. (C) Butterfly plots for the IED class obtained using WC. The colored traces (blue) represent the individual events, the thick black lines the mean traces and grey lines, the standard error. The CED (Visual class 1) were not classified using *Wave_clus*, but were included in GLM2 as a separate effect of interest. (D) IED-related BOLD maps for the IZ1 WC class, superimposed on the patient's co-registered postoperative structural MRI. The map's concordance was discordant. The map for VC1 in GLM2 is identical to the one shown in (B). Cross-hair placement: for the concordant maps, within one of the BOLD clusters within, or overlapping with, the EZ, for discordant maps: within the EZ.

*Visual classification*: Reviewer H1 classified the IEDs into 3 classes, two of which were labelled IZ1 (classes 1 and 2) comprising a total of 1035 IEDs; class 1 consisted of focal IEDs (*N* = 770) with (CED pattern of >1 per sec), and class #2 consisted of regional IEDs (*N* = 265); see Fig. 5.

*Automated classification*: The CED pattern (visual class 1) was not classified using WC, but included in GLM2. WC classified the remaining 313 IEDs into three classes, one of which was labelled as IZ1 (class 1), consisting of focal 75 IEDs.

###### 3.2.1.4.2 BOLD results

*GLM1*: the concordances for the two IZ1 classes were: *D* for class 1 and *C* for class 2. For class 1 the global maximum located in the right lateral occipital lobe and in the EZ for class 2.

*GLM2*: the degree of concordance for the two IZ1 classes (Visual class 1 and WC class 1) was *D*. For Visual class 1 the global maximum was located in the posterior cingulate cortex and for WC class 1 the global maximum was located in the left posterior temporal lobe.

## 4 Discussion

Our aim was to determine whether classifying intracranially recorded IEDs with simultaneous fMRI using an automated spike classification algorithm could provide more biologically meaningful IED-related BOLD maps compared to those obtained following visual expert analysis in a group of patients with well characterised EZ.

To do this, IED-related BOLD maps obtained based on the automated algorithm were compared to the visual (classic/standard) classification (along the lines of our previous work on scalp EEG-fMRI; Pedreira et al., 2014) in terms of the presence or absence of BOLD changes in the EZ. To facilitate interpretation of the findings, we limited this analysis to the IEDs observed within the EZ (IZ1 IEDs) since we can be more confident of the location of their generators than the propagation IEDs.

One of the main challenges associated with the use of icEEG data for fMRI mapping is the difficulty of forming a parsimonious model of potential BOLD changes that reflects the complex spatio-temporal dynamics of IEDs. We found that the IED-related BOLD maps were concordant to the area of resection for 83% of the IZ1 classes for the automated algorithm compared to 75% for the visual classes (see Table 3). At the single-subject level, we found improved concordance in four patients using the automated algorithm, no change in concordance between the automated and visual classification in three patients and a worse degree of concordance in one. These results suggest that the automated classification of IEDs using WC on icEEG recordings can be used to create a more biologically meaningful model of the associated hemodynamic changes in some patients, with an acceptable risk of obtaining more spurious results, in addition to helping circumvent the problem of subjective (see Sharma et al., 2017) and time-consuming visual classification of IEDs.

### 4.1 Clinical and biological significance

To date, there have only been three studies focusing on the whole-brain mapping of IED-related BOLD changes using simultaneous icEEG-fMRI (Vulliemoz et al., 2011; Cunningham et al., 2012; Aghakhani et al., 2015). It is important to note that in our centre, the main motivation for the work based on icEEG-fMRI (Ridley et al., 2007; Vulliemoz et al., 2011; Chaudhary et al., 2016; Murta et al., 2016) is to gain a better understanding of the neurobiology of epilepsy, rather than to evaluate it as a potential clinical tool. Therefore, we propose that the present work's potential clinical significance lies mostly in its putative impact on the classification of IED in intracranial recordings; As explained previously and discussed further below, here the primary role of the simultaneously-acquired fMRI data is in providing a basis for the comparison of two types of IED classification.

In previous icEEG-fMRI studies of IED, these were detected and classified by EEG reviewers (Vulliemoz et al., 2011; Cunningham et al., 2012; Aghakhani et al., 2015). However, our previous study confirmed that the classification of icEEG IEDs can be inconsistent between EEG reviewers and that the automated classifications obtained using *Wave_clus* fall within inter-observer variability (Sharma et al., 2017). Furthermore, we also previously demonstrated that the same algorithm, when applied to scalp EEG, showed an improved fMRI localisation compared to visual classification as 72% of the BOLD changes associated with WC classification were concordant to the presumed irritative zone (compared to the 50% of the visual classes) (Pedreira et al., 2014). Therefore, although WC classifications of IEDs are statistically indistinguishable from the human ones (Sharma et al., 2017), the results from Pedreira et al. (2014) indicate that the statistical analysis underlying WC classification may yet reveal a specific capability of classifying IEDs that is different from humans. Therefore, in the absence of a ground truth in terms of IED classification, and similar to Pedreira et al. (2014), we sought to compare IED classification through independent data, namely the associated BOLD changes and in particular their relationship with the EZ.

Previous icEEG-fMRI studies involved in mapping IED-related BOLD across the whole brain assessed concordance based on location of a significant BOLD cluster to the most active spiking electrode contact (Vulliemoz et al., 2011; Aghakhani et al., 2015). The largest study carried out by Aghakhani et al. (2015) speculated that discordant (BOLD changes outside the lobe of the most active electrode) IED-related BOLD changes was due to the EZ being located elsewhere as this finding was found in patients with a poor postsurgical outcome (Aghakhani et al., 2015). This is consistent with previous scalp EEG-fMRI studies that indicate the absence of statistically significant BOLD changes in the area of resection can be predictive of a poor postsurgical outcome (Thornton et al., 2010, An et al., 2013, Coan et al., 2015; Centeno et al., 2017). Therefore, we assessed the presence of IZ1 IED-related BOLD changes using the best available gold standard defined as the area of resection in good postsurgical outcome patients (Grouiller et al., 2011).

In this study, any significant BOLD cluster was labelled concordant if it overlapped or was within 2 cm of (within the same lobe as) the area of surgical resection (Coan et al., 2015). Some previous scalp EEG-fMRI studies on the clinical relevance of IED mapping in the pre-surgical evaluation of patients with severe epilepsy have focused on the use of the location of the statistically most significant BOLD cluster, the global maximum (GM), as the putative marker of the EZ (Moeller et al., 2009; Thornton et al., 2010; Pittau et al., 2012; An et al., 2013). This is mainly based on two considerations: the cluster containing the statistical maximum is unique for any given map, which is a convenient simplification, and it represents the most likely location of the generator by some, albeit indirect, measure of the statistically strongest haemodynamic change. However, the sensitivity of the GM in localising the EZ is still inconclusive, as some studies have shown that it localises the EZ (defined as the area of resection in good postsurgical outcome patients) in less than half of the patients (Grouiller et al., 2011, An et al., 2013; Centeno et al., 2017). As mentioned previously, we defined concordance similar to Coan et al. (2015), as the presence of any significant BOLD cluster within or close to the EZ, and who showed that the presence of a significant BOLD cluster within or in close proximity to the EZ is strongly associated with good outcome (and absence of any cluster in the EZ, with a poor outcome). Indeed, in the present study only a small percentage of IED datasets showed the GM to be within or in the immediate proximity of the EZ for BOLD maps obtained using the visual and automated approach (16% and 17% respectively) (see Table 3) raising further questions on the notion of the global maximum as a marker of EZ. Furthermore, as discussed under *Methodological considerations*, there are physical reasons why the choice of the GM as part of the concordance assessment may be suboptimal for icEEG-fMRI data. The most important property of any BOLD-based assessment scheme is that provides an even playing field based on the spatial relationship between regions of BOLD changes and the epileptogenic generators.

In this study, four patients showed an improved concordance following automated IED classification (patients 4, 5, 7 and 8). As mentioned previously, our main focus was comparing concordance levels for the classes obtained by WC and visual classification and not comparing the spatial distribution of the EEG classes themselves. However, the advantage of using our automated approach can be seen for patient 5 for example (see Fig. 5). In this patient, the EEG reviewer and WC identified two IZ1 IED classes involving similar electrodes with same distribution: one with a focal distribution involving the FP electrodes and the second with a regional distribution involving FP and AM electrodes (see Table 3 and Fig. 3). Close examination of the two classifications shows that WC identified more IEDs for both classes (FP-focal: *N =* 333, FP-AM regional: *N =* 591) compared to the visual classification (FP-focal: *N =* 142, FP-regional: *N =* 81). Examination of Supplementary Table 1 shows that considerably more IEDs were classified as IZ2 IEDs for the visual classification (*N =* 917) whereas WC classified less IEDs in IZ2 (*N =* 202) (see Supplementary Table 1) indicating that perhaps the visual classification resulted in IEDs that were more mixed between IZ1 and IZ2. Given the BOLD result is concordant for the FP-focal class for WC classification (compared to a discordant result for the visual classification), this may suggest that the IEDs were more accurately classified using WC.

### 4.2 Methodological considerations and future work

The WC algorithm is based on the statistical characterisation of the entire IED population (in any given recording), and therefore it differs quite fundamentally from human expert classification, which is mostly done in sequence and focused on individual events, with a certain degree of consideration of the rest of the IED population. This is a way of integrating inherent patient-specific characteristics into the expert assessment, along with more general knowledge; in the statistical approach, the statistical weight of any feature (number of events, size of the difference with the rest of the sample) is factored in a more global and objective/categorical way. This means that it is possible that certain statistically minor (sparse) events, which may be deemed clinically important by any given expert, may be overlooked by the WC feature extraction process.

Our automated classification pipeline was designed for the automated classification of individual IEDs as reflected in its use of a fixed-length time window centred around the peak of the IED to incorporate the maximum information of the IEDs with smallest possible time span to minimise the impact of noise (Sharma et al., 2017). Therefore it is not suitable in its current form to be applied to the EEG patterns with a fast repetitive nature such as polyspikes, PFA and very continuous CEDs; commonly observed EEG patterns in FCD patients (Palmini et al., 1995; Turkdogan et al., 2005; Widdess-Walsh et al., 2007). Therefore, to ensure that our BOLD models were as statistically efficient as possible, we included a manual step in our classification using *Wave_clus*. For example, regarding CEDs the EEG reviewer made a note of the frequency of the CEDs and if they occurred >1 per second, they were not automatically classified but modelled in the GLM; this was carried out in two patients (patients 3 and 8) (see Fig. 5 for an example for patient 3). The automated classification of IEDs for patient 3 resulted in more discordant maps than the visual classification (see Table 3 and Fig. 5). We note that uniquely for this patient the channels of interest, which were selected entirely based on information gained from the clinical report, included those showing frequent CEDs (channels DA3, DA4; see Fig. 5 and Table 2). Therefore, the continuous nature of this EEG pattern, which the algorithm is not designed to handle, may have resulted in sub-optimal classification of the individual IEDs.

The loss of MR signal in the vicinity of the electrodes in our data dataset due to the susceptibility artefact (Carmichael et al., 2012) is likely to result in reduced statistical power to detect local BOLD changes, which may partly explain why the GM was found to be distant to the EZ in some cases, providing further justification for the concordance scheme used here.

However, one of the main potential advantages of icEEG-fMRI compared to scalp EEG-fMRI is the opportunity to map the haemodynamic response associated with very focal discharges, as observed on depth EEG for example. In this study we defined focal discharges as IEDs occurring in up to 4 contiguous electrode contacts. The IED-related BOLD maps acquired using WC show that 87% of the WC IZ1 focal IEDs (13/15) show significant BOLD changes in the EZ as well as distant from the EZ (see Supplementary Table 3 and Fig. 3 for an example). These findings show that even for IEDs that are classified as being focal, distant regions maybe involved in their generation. Therefore, our results further reinforce the notion that even for very focal discharges, the epileptic network can be widespread, involving regions responsible for generating the IED and seizures, and regions remote from this area.

The practical benefits of our approach include time saving and dispensation of the need for reproducibility studies (Flanagan et al., 2009) and therefore, the uncertainty associated with changes in human EEG rater marking. Similar to previous studies using WC (Pedreira et al., 2014; Sharma et al., 2017) this study focused on the classification of IEDs detected by an EEG reviewer. In this regard it would be useful to also automate the detection of IEDs and then classify the markings using WC.

In this work, we chose to map the BOLD changes associated with each IED class separately, even though it may have been possible to combine some classes as a single effect of interest based on their field distributions and morphologies: essentially, it is conceivable that some classes represent variations in the activity of a single generator (for example, all the IED recorded in the left temporal lobe of patient 4). However, this is a complex issue that belongs more specifically to the field of fMRI modelling and which we have previously studied for IED recorded on the scalp during fMRI (Salek-Haddadi et al., 2006).

The application of our technique to a group of patients with varied surgical outcome would allow us to better study the biological of the observed BOLD patterns. Such studies could also include a consideration of their relationship with the seizure onset zone, in addition to that with the EZ, which is generally more extensive, and perhaps shaed additional light on their associated brain networks.

## 5 Conclusion

We found automated IED classification on icEEG data recorded during fMRI to be feasible and to result in IED-related BOLD maps that may contain similar or greater biological meaning compared to the conventional approach in the majority of the cases studied. Furthermore, we have shown that the BOLD changes associated with even very localised IEDs on icEEG, can be widespread. Considering the additional putative advantages of automated classification (increased time efficiency and reduced impact of human EEG interpretation), we anticipate that this tool may provide new insights into the regions responsible for generating IEDs.
