# Supplementary material for: BOLD mapping of human epileptic spikes recorded during simultaneous intracranial EEG-fMRI: The impact of automated spike classification
Source: Neuroimage. 2019 Jan 1;184:981–92. doi: 10.1016/j.neuroimage.2018.09.065 (PMC6264381; doi:10.1016/j.neuroimage.2018.09.065)
Supplement: Supplementary data [file mmc1.docx]

Supplementary Table 1. IED classification results for the visual and *Wave_clus* methods. Cells shaded in grey show the visual classes (CED with > 1 IED/sec) included in BOLD model GLM2.

| **Visual** | | | | | | | | | ***Wave_clus*** | | | | | | | | |
| --- | --- | --- | --- | --- | --- | --- | --- | --- | --- | --- | --- | --- | --- | --- | --- | --- | --- |
| **Patient** | **Classification** | | | **Temporal pattern** | | | **IZ** | **IED Field Distribution** | **Patient** | **Classification** | | | **Temporal pattern** | | | **IZ** | **IED Field Distribution** |
|  | **IED Classes** | **# IEDs** | **Total # IEDs** | **SED** | **CED** | **PFA** |  |  |  | **IED Classes** | **# IEDs** | **Total # IEDs** | **SED** | **CED** | **PFA** |  |  |
| **1** | VC1: G4,5 | 70 | 590 | * |  |  | IZ1 | Focal | **1** | WCC1: G23 | 127 | 488 | * |  |  | IZ1 | Focal |
|  | VC2: G12-15 | 30 |  | * |  |  | IZ1 | Focal |  | WCC2: G4_5_13_21_29_DP | 131 |  | * |  |  | IZ1 | Regional |
|  | VC3: G4-6 + G12,13 +G22-24 + G28-30 | 60 |  | * |  |  | IZ1 | Regional |  | WCC3: G4_5_29 | 78 |  | * |  |  | IZ1 | Non-contiguous |
|  | VC4: G12-15 + G21-24 + DP2-4 | 218 |  | * |  |  | IZ1 | Regional |  | WCC4: G13_20_21_DP | 152 |  | * |  |  | IZ1 | Focal |
|  | VC5: G4-8 +G12-15 + G20-24 + G28-30 + DP2-4 | 212 |  | * |  |  | IZ1 | Widespread | **2** | WCC1: D4_5 | 498 | 760 |  | * |  | IZ1 | Focal |
| **2** | VC1: DA3-6 | 423 | 892 |  | * |  | IZ1 | Focal |  | WCC2: D4_5_GA50_51_52 | 106 |  | * |  |  | IZ1 | Regional |
|  | VC2: DA4,5 + GA51 | 261 |  | * |  |  | IZ1 | Focal |  | WCC3: D4_5_GA51 | 156 |  | * |  |  | IZ1 | Focal |
|  | VC3: DA2-6 + GA49-54 | 208 |  | * |  |  | IZ1 | Regional | **3** | VC1: DA3_4 | 770 | 1083 |  | * |  | IZ1 | Focal |
| **3** | VC1 DA3,4 | 770 | 1035 |  | * |  | IZ1 | Focal |  | WCC1: DA3_4_G1 | 75 |  | * |  |  | IZ1 | Focal |
|  | VC2: DA3,4 + G1 18,27,35,43 | 265 |  | * |  |  | IZ1 | Regional |  | WCC2: G2_5 | 153 |  | * |  |  | IZ2 | Focal |
|  | VC3: G2 6,14 | 195 |  | * |  |  | IZ2 | Focal |  | WCC3: G2_5_6_14_15 | 85 |  | * |  |  | IZ2 | Focal |
| **4** | VC1: LAH1,2 + LPH1,2 + LA3,4 | 60 | 1216 | * |  |  | IZ1 | Regional | **4** | WCC1: LA2_LAH1_2 | 360 | 1062 | * |  |  | IZ1 | Focal |
|  | VC2: LAH1-2 | 359 |  | * |  |  | IZ1 | Focal |  | WCC2: LAH1_2_LPH1 | 45 |  | * |  |  | IZ1 | Focal |
|  | VC3: LA3-4 | 57 |  | * |  |  | IZ1 | Focal |  | WCC3: RA1_2 | 549 |  | * |  |  | IZ2 | Focal |
|  | VC4: LPH1-2 | 96 |  | * |  |  | IZ1 | Focal |  | WCC4: RA1-2 RH1 LH1-2-LPH1 (only polyspikes) | 41 |  | * |  |  | IZ2 | Non-contiguous |
|  | VC5: RA1,2 + RH1,2 | 634 |  | * |  |  | IZ2 | Focal | **5** | WCC1: FP4 | 333 | 1126 | * |  |  | IZ1 | Focal |
|  | VC6: RA1,2 + RAH1,2 +LAH2,3 + LPH2,3 | 10 |  | * |  |  | IZ2 | Non-contiguous |  | WCC2: AM2-4 + FP1-4 | 591 |  | * |  |  | IZ1 | Regional |
| **5** | VC1: FP2-4 | 142 | 1140 | * |  |  | IZ1 | Focal |  | WCC3: AM_FP_PMFG_IFG | 202 |  | * |  |  | IZ2 | Non-contiguous |
|  | VC2: FP2-4 + AM2-4 | 81 |  | * |  |  | IZ1 | Regional | **6** | WCC1: ASMA1-2 + PSMA 2-3 | 364 | 795 | * |  |  | IZ1 | Focal |
|  | VC3: AM2-4 + FP1-4 + PMFG3-6 + IFG9-11 | 203 |  | * |  |  | IZ2 | Non-contiguous |  | WCC2: PSMA 2-3 | 250 |  | * |  |  | IZ1 | Focal |
|  | VC4: AM1-4 + FP3-4 + FP1-8 + AM1-14 + ASMA2-5 + PMFG3-10 + IFG5-10 | 296 |  | * |  |  | IZ2 | Non-contiguous |  | WCC3: PC4-5 + AI5-6 | 181 |  | * |  |  | IZ2 | Non-contiguous |
|  | VC5: FP1-4 + AM1-6 + FP1-8 + AM1-14 + FOF1-10 + ASMA2-7 + PMFG4-12 + IFG5-11 | 418 |  | * |  |  | IZ2 | Non-contiguous | **7** | WCC1: SF_GB | 68 | 277 | * |  |  | IZ1 | Non-contiguous |
| **6** | VC1: PSMA1-3 | 211 | 1033 | * |  |  | IZ1 | Focal |  | WCC2: SF6_7 | 177 |  | * |  |  | IZ1 | Focal |
|  | VC2: ASMA1-3 | 46 |  | * |  |  | IZ1 | Focal |  | WCC3: SF8 | 32 |  | * |  |  | IZ1 | Focal |
|  | VC£: ASMA1-3 + PSMA1-3 | 476 |  | * |  |  | IZ1 | Regional | **8** | VC2: D2_3_4 | 2481 | 3246 |  | * |  | IZ1 | Focal |
|  | VC4: PC5,6 | 150 |  | * |  |  | IZ2 | Focal |  | WCC1: G38-39-40-46-47 | 256 |  | * |  |  | IZ1 | Regional |
|  | VC5: PC5,6 + AI5,6 | 150 |  | * |  |  | IZ2 | Non-contiguous |  | WCC2: D1_3_4 G37-38-39 | 184 |  | * |  |  | IZ1 | Regional |
| **7** | VC1: SF5-7 | 168 | 755 | * |  |  | IZ1 | Focal |  | WCC3: D1_3_4 | 75 |  | * |  |  | IZ1 | Focal |
|  | VC2: GB4-6 + 14-16 | 90 |  | * |  |  | IZ1 | Regional |  | WCC4: G45-46 | 128 |  | * |  |  | IZ1 | Focal |
|  | VC3: GC5-16 | 474 |  | * |  |  | IZ1 | Regional |  | WCC5: D1_3_4 G22_23_29_30_31_37_38 | 66 |  | * |  |  | IZ1 | Regional |
|  | VC4: SF5-7 + GB5-8 + GC5,10,11,12,15,16 | 23 |  | * |  |  | IZ1 | Non-contiguous |  | WCC6: D1_3_4 G22_23_30_31_38_39_40 | 56 |  |  |  | * | IZ1 | Widespread |
| **8** | VC1: D1 | 43 | 3567 | * |  |  | IZ1 | Focal |  | | | | | | | | |
|  | VC2: D2 | 2481 |  |  | * |  | IZ1 | Focal |  |  |  |  |  |  |  |  |  |
|  | VC3: G23 | 83 |  | * |  |  | IZ1 | Focal |  |  |  |  |  |  |  |  |  |
|  | VC4: G31 | 72 |  | * |  |  | IZ1 | Focal |  |  |  |  |  |  |  |  |  |
|  | VC5: G36 | 209 |  | * |  |  | IZ1 | Focal |  |  |  |  |  |  |  |  |  |
|  | VC6: G38 | 226 |  | * |  |  | IZ1 | Focal |  |  |  |  |  |  |  |  |  |
|  | VC7: G44 | 140 |  | * |  |  | IZ1 | Focal |  |  |  |  |  |  |  |  |  |
|  | VC8: G45 | 127 |  | * |  |  | IZ1 | Focal |  |  |  |  |  |  |  |  |  |
|  | VC9: G47 | 124 |  | * |  |  | IZ1 | Focal |  |  |  |  |  |  |  |  |  |
|  | VC10: Widespread D1-D2 | 62 |  |  |  | * | IZ1 | Widespread |  |  |  |  |  |  |  |  |  |

Abbreviations: SED: single isolated epileptiform discharge; CED: continuous epileptiform discharges, PFA: paroxysmal fast activity. VC: visual class; WCC: Wave_clus (WC) class

Supplementary Table 2. GLM1 IZ1 IED-related BOLD results. The GLM1 model of the BOLD changes was built based on the results of visual IED classification.

| **Patient** | **IED class effects of interest** | **Concordant?** | **Summary of BOLD in EZ** | | | **Summary of BOLD in rest of the brain** | |
| --- | --- | --- | --- | --- | --- | --- | --- |
|  |  |  | **# voxels in EZ** | **Max z-score in EZ** | **HRF sign of peak change** | **GM localization** | **BOLD localization** |
| **1** | VC1: G4,5 | Y | 19 | 4.21 | Negative | R H | R Cer, P-T, O, P T, S Par  L IFG |
|  | VC2: G12-15 | Y | 51 | 4.9 | Negative | M O | R SMA, M Par, S Par, IFG, P T, I Par, R T pole  L IFG, P T |
|  | VC3: G4-6 + G12,13 +G22-24 + G28-30 | Y | 9 | 4.19 | Negative | L IFG | R Cer, S Par, IFG, H  L, H, C, P-T |
|  | VC4: G12-15 + G21-24 + DP2-4 | Y* | 32 | 5.01 | Negative | --- | L SMA, P-T, IFG  R S Par |
|  | VC5: G4-8 +G12-15 + G20-24 + G28-30 + DP2-4 | Y* | 91 | 4.50 | Positive | --- | R SFG, P-T, O, H, IFG  L, O |
| **2** | VC1: DA3-6 | Y | 34 | 4.05 | Positive | R P-T | R I Par, IFG, H, O, MFG P CC  L T pole, O, P-T, H, S Par |
|  | VC2: DA4,5 + GA51 | N | - | - | - | L M O | R M Par, T pole  L H, P-T, M PFC, M Par |
|  | VC3: DA2-6 + GA49-54 | Y | 8 | 4.21 | Negative | R I Par | R F-T, H, P T, T pole  L P T |
| **3** | VC1: DA3,4 (770) | N | - | - | - | R O | R P T, P CC  L Thal |
|  | VC2: DA3,4 + G1 18,27,35,43 (265) | Y* | 29 | 4.67 | Positive | --- | --- |
| **4** | VC1: LAH1,2 + LPH1,2 + LA3,4 | Y | 133 | 4.83 | Positive | L O | R IFG, SFG, MFG, P T, O, I Par, S Par  L IFG, SFG, I Par, P T, SMA, P CC, M O |
|  | VC2: LH1-2 | Y | 330 | 5.20 | Positive | L O | R, IFG, PFC, H, O, S Par, I Par, M Par  L MFG, M Par, SMA, S Par, CC, PFC |
|  | VC3: LA3-4 | N | - | - | - | R SFG | R MFG, H, F-T, Cer, M Par,  L IFG, S Par |
|  | VC4: LP1-2 | N | - | - | - | L OFC | --- |
| **5** | VC1: FP2-4 | Y | 27 | 3.98 | Negative | R M O | R H, MFG, O, Thal, A CC, IFG, I Par, S Par,  L, H, T pole, S Par, T pole, MFG, IFG, P T |
|  | VC2: FP2-4 + AM2-4 | N | - | - | - | R M O | R M O, I Par, M Par, MFG, Cer, SFG  L M O, OFC, M Par |
| **6** | VC1: PSMA1-3 | Y | 21 | 4.01 | Negative | L OFC | L MFG |
|  | VC2: ASMA1-3 | Y | 231 | 6.11 | Negative | R IFG | R I Par, P T  L T pole, S Par, IFG, MFG |
|  | VC3: ASMA1-3 + PSMA1-3 | Y | 32 | 3.8 | Positive | R F-T | R M Par  L O, P T, F-T, M O |
| **7** | VC1: SF5-7 | N | - | - | - | L P T | R P T, OFC, P CC |
|  | VC2: GB4-6 + 14-16 | Y | 5 | 3.51 | Positive | L M O | R OFC, IFG, P T, M O, S Par, MFG, SFG, A CC SMA  L P T, M P |
|  | VC3: GC5-16 | Y | 7 | 3.55 | Negative | R OFC | R I Par, Thal, SFG, CC  L T pole, P T |
|  | VC4: SF5-7 + GB5-8 + GC5,10,11,12,15,16 | Y | 6 | 3.28 | Negative | L M O | R IFG, M Par, O  L S Par, M O, SMA, IFG |
| **8** | VC1: D1 | Y | 124 | 3.88 | Negative | R IFG | R M O, SFG, MFG, P T |
|  | VC2: D2 | Y* | 200 | 5.19 | Positive | --- | R T pole, O  L IFG, I Par, P T, SMA, Thal, O |
|  | VC3: G23 | Y | 56 | 5.00 | Negative | R IFG | R PFC, SFG, SMA, OFC, M O, P T, I Par  L O, MFG, OFC |
|  | VC4: G31 | Y | 6 | 3.50 | Positive | R P T | R M PFC  L I Par, OFC, S Par |
|  | VC5: G36 | Y | 122 | 5.09 | Positive | R IFG | R M O, T Pole  L IFG, S Par, SMA, IFG, I par, S par, thal |
|  | VC6: G38 | Y* | 451 | 5.37 | Negative | --- | R PFC, MFG, SMA, SFG  L, IFG, PFC, I Par, M Par, CC |
|  | VC7: G44 | N | - | - | - | L MFG | R H, P CC  L P T, F-T |
|  | VC8: G45 | Y | 46 | 4.16 | Positive | R M PFC | R M O, IFG  L, I Par, H, P T, F-T, SFG, I Par |
|  | VC9: G47 | N | - | - | - | L PFC | R MFG, OFC  L I Par, M Par, T pole, IFG |

Abbreviations: L: left; R: right; H: Hippocampus; A: anterior; P: posterior; Par: parietal; P-T: parieto-temporal; Cer: Cerebellum; O: occipital; SMA: supplementary motor area; IFG: inferior frontal gyrus; MFG: middle frontal gyrus; SFG: superior frontal gyrus; M: mesial; CC: cingulate cortex; PFC: prefrontal cortex; S: superior; I: inferior; T: temporal; T-O: temporo-occipital; F-T: fronto-temporal; C: caudate; Thal: Thalamus; OFC: orbitofrontal cortex; GM: global maximum; * = BOLD cluster in EZ is the global maximum

Supplementary Table 3. GLM2 IZ1 IED-related BOLD results. The GLM2 model of the BOLD changes was built based on the results of *Wave_clus* IED classification; for patients 3 and 8, one of the effects of interest was taken from the visual classification (see Supplementary Table 1).

| **Patient** | **IED class effects of interest** | **Concordant?** | **Summary of BOLD in EZ** | | | **Summary of BOLD in rest of the brain** | |
| --- | --- | --- | --- | --- | --- | --- | --- |
|  |  |  | **No. of Voxels in EZ** | **Max z-score in EZ** | **HRF sign of peak change** | **GM localization** | **BOLD localization** |
| **1** | WCC1: G23 | Y | 180 | 5.79 | Negative | R O | R Par  L F Pole, T-P, O |
|  | WCC2: G4_5_21_29_DP | Y | 10 | 3.82 | Positive | L P T | R T, O |
|  | WCC3: G4_5_29 | Y* | 19 | 4.05 | Positive | --- | R T, MFG |
|  | WCC4: G13_20_21_DP | Y | 7 | 3.95 | Positive | L H | R H, O  L, O, P T |
| **2** | WCC1: D4_5 | Y | 5 | 3.56 | Negative | R P T | R H, SFG, OFC, F Pole  L T pole, SFG, Thal, S Par |
|  | WCC2: D4_5_GA49_51 | N | - | - | - | L H | R F-T, S Par  L H, P T |
|  | WCC3: D4_5_GA51 | Y | 9 | 3.72 | Positive | R A P | R SMA, T-P, thal, F pole, T pole  L P T, IFG |
| **3** | VC1: DA3_4 | N | - | - | - | P CC | R S Par, F Pole  L thal |
|  | WCC1: DA3_4_G1 | N | - | - | - | L P T | R H, S Par  L O |
| **4** | WCC1: LA2_LH1_2 | Y | 5 | 3.54 | Positive | CC | R M Par, MFG, OFC  L MFG, IFG, Par, P CC, O |
|  | WCC2: LH1_2_LP1 | Y* | 21 | 4.54 | Positive | --- | R A Par  L OFC, M Par, O, SMA, A Par, P CC |
| **5** | WCC1: FP4 | Y | 269 | 5.63 | Positive | R O | R H, P T, S Par, O, I Par, MFG  L IFG, S Par, A T |
|  | WCC2: AM_FP | Y | 155 | 4.46 | Positive | L S Par | R H, P-T, M Par, SFG, T Pole  L I Par, T pole, MFG, H, PFC, SFG, IFG |
| **6** | WCC1: ASMA_PSMA | Y | 130 | 4.75 | Negative | L F pole | R F pole, O, SFG, T, S Par, H,  L M Par, MFG, P T, F-T |
|  | WCC2: PSMA | Y | 9 | 3.59 | Negative | R P T | L F-T, I Par, SFG |
| **7** | WCC1: SF_GB | Y | 26 | 4.02 | Positive | L O | R O, S Par, SFG, IFG  L, F-T, SMA, IFG |
|  | WCC2: SF6_7 | Y | 39 | 4.03 | Negative | R S Par | R I Par, T-O, SFG, MFG, I Par, T pole, H  L PFC, F-T |
|  | WCC3: SF8 | Y | 12 | 3.81 | Positive | R T pole | R MFG, IFG  L S Par, F-T, O P CC |
| **8** | VC2: D2_3_4 | Y* | 180 | 5.6 | Positive | --- | R F-T, O, A CC, F pole  L, T-P, S Par, M Par, P T |
|  | WCC1: G38-39-40-46-47 | Y* | 40 | 4.26 | Positive | --- | R O  L OFC, O, M Par |
|  | WCC2: D1_3_4 G37-38-39 | Y | 595 | 5.64 | Negative | R S Par | R F pole, IFG, SFG, T pole, H, P CC, SMA  L F-T, IFG, T pole, T-O |
|  | WCC3: D1_3_4 | Y* | 22 | 4 | Positive | --- | R S Par  L M Par, OFC |
|  | WCC4: G45-46 | N | - | - | - | L S Par | R OFC, F-T  L M Par, F-T, CC, SMA |
|  | WCC5: D1_3_4 G22_23_29_30_31_37_38 | Y | 613 | 5.39 | Negative | R F-T | R P T,  L T-P, M Par, MFG, SFG |

Abbreviations: L: left; R: right; H: Hippocampus; A: anterior; P: posterior; Par: parietal; P-T: parieto-temporal; Cer: Cerebellum; O: occipital; SMA: supplementary motor area; IFG: inferior frontal gyrus; MFG: middle frontal gyrus; SFG: superior frontal gyrus; M: mesial; CC: cingulate cortex; PFC: prefrontal cortex; S: superior; I: inferior; T: temporal; T-O: temporo-occipital; F-T: fronto-temporal; C: caudate; Thal: Thalamus; OFC: orbitofrontal cortex; GM: global maximum; VC: Visual class; WCC: Wave_Clus class.

* = BOLD cluster in EZ is the global maximum
